# Characterization and phylogenetic analysis of the complete Green-winged macaw (*Ara chloropterus*) mitogenome

**DOI:** 10.1080/23802359.2019.1711229

**Published:** 2020-01-16

**Authors:** Da-wei Liu, Yang Pan, Jian He, Jing Zhou, Chun-ping Xie

**Affiliations:** a Nanjing Forest Police College, Nanjing, China;; b Forensic Identification Center for Forest Police of the State Forest Bureau, Nanjing, China;; c Key Laboratory of State Forest and Grassland Administration Wildlife Evidence Technology, Nanjing, China;; d Ministry of Ecology and Environment, Nanjing Institute of Environmental Sciences, Nanjing, China

**Keywords:** Green-winged macaw (Ara chloropterus), mitochondrial genome, phylogenetic analysis

## Abstract

In this study, we sequenced and characterized the complete mitochondrial genome of Green-winged macaw (*Ara chloropterus*) listed on CITES Appendix II. The circular double-stranded genome was 16,991 bp in size and included 22 transfer RNA (tRNA) genes, 13 protein-coding genes (PCGs), two ribosomal RNA genes (*rrnL* and *rrnS*), and one non-coding control region (D-loop). Both order and arrangement of genes were identical to those of other animal mitogenomes. The base content was 30.00% A, 14.38% G, 23.27% T, and 32.35% C, with an A + T content of 53.37%. Furthermore, phylogenetic analysis indicated that *A. chloropterus* is closely related to the Blue and yellow macaw (*A. ararauna*).

The Green-winged macaw (*Ara chloropterus*) is a kind of large *Ara* macaws (Forshaw 2010), which belongs to subfamily Arinae (Neotropical Parrots). Although this bird is listed as of least concern, the population is currently decreasing as a result of ongoing habitat destruction and unsustainable levels of exploitation (BirdLife International 2019). In order to better protect the species, *A. chloropterus* was listed in CITES Appendix II, owing to its association with wild animal trade. Herein, the complete mitogenome of *A. chloropterus* was determined.

A feather sample was collected from Hongshan Zoo (N32°09′, E118°08′). The voucher specimen (A-2019003) was stored in the Key Laboratory of Wildlife Evidence Technology State Forest and Grassland Administration. Total DNA was extracted using a Universal Genomic DNA Extraction Kit (Takara, Beijing, China). PCR primers were designed based on known sequences of *A. ararauna* (GenBank accession no. KF010315.1) and *A. glaucogularis* (GenBank accession no. JQ782215.1). PCR-amplified fragments were subjected to Sanger sequencing and then assembled into a complete mitogenome sequence, submitted to GenBank (accession no. MN604694).

As in other multicellular animals (Bridge et al. 1992), the double-stranded mitogenome of *A. chloropterus* was circular, ∼16 kb in length (16,991 bp to be exact), and contained two ribosomal RNA genes (*rrnL* and *rrnS*), 13 protein-coding genes (PCGs), 22 transfer RNA (tRNA) genes, and a single non-coding control region. Meanwhile, the gene arrangement of the species’ mitogenome was identical to that of other birds (Lan et al. 2019; Liu et al. 2019).

Furthermore, the nucleotide composition of the mitogenome was 30.00% A, 14.38% G, 23.27% T, and 32.35% C, with an A + T content of 53.37%. Among the PCGs, *nad5* (1809 bp) and *atp8* (168 bp) were the longest and shortest, respectively, and ATG was the most prevalent start codon, whereas TAA was the most prevalent stop codon. The lengths of *rrnL* and *rrnS* were 972 and 1567 bp, respectively. The two genes were located in between *trnF* and *trnL2* and were separated by *trnV*. In addition, the *A. chloropterus* mitogenome contained 22 tRNAs, which ranged in size from 66 to 76 bp, interspersed throughout the genome. In *A. ararauna*, the 1487 bp D-loop was located between *trnE* and *trnF*, and the A + T content (57.70%) was higher than the G + C content (42.30%) and the full sequence A + T content (53.37%).

In order to investigate the phylogenetic relationships between *A. chloropterus* and other parrot species, we constructed a neighbour-joining phylogenetic tree using MEGA 7.0 under the Kimura 2-parameter model with 1000 bootstrap replicates (Kumar et al. 2016). The analysis was based on the whole mitogenome sequences of *A. chloropterus*, 14 other species from Order Psittaciformes, and 2 species from Order Passeriformes, which were used as outgroups. The topologies of molecular phylogenetic analysis showed that target species *A. chloropterus* was placed as sister to *A. ararauna*, which, together, were closely related to other macaws, namely, *Primolius couloni* and *Orthopsittaca manilata* (Figure 1). The present study generated the complete mitogenome of *A. chloropterus* and provides fundamental genetic information for the species’ conservation.

**Figure 1.**
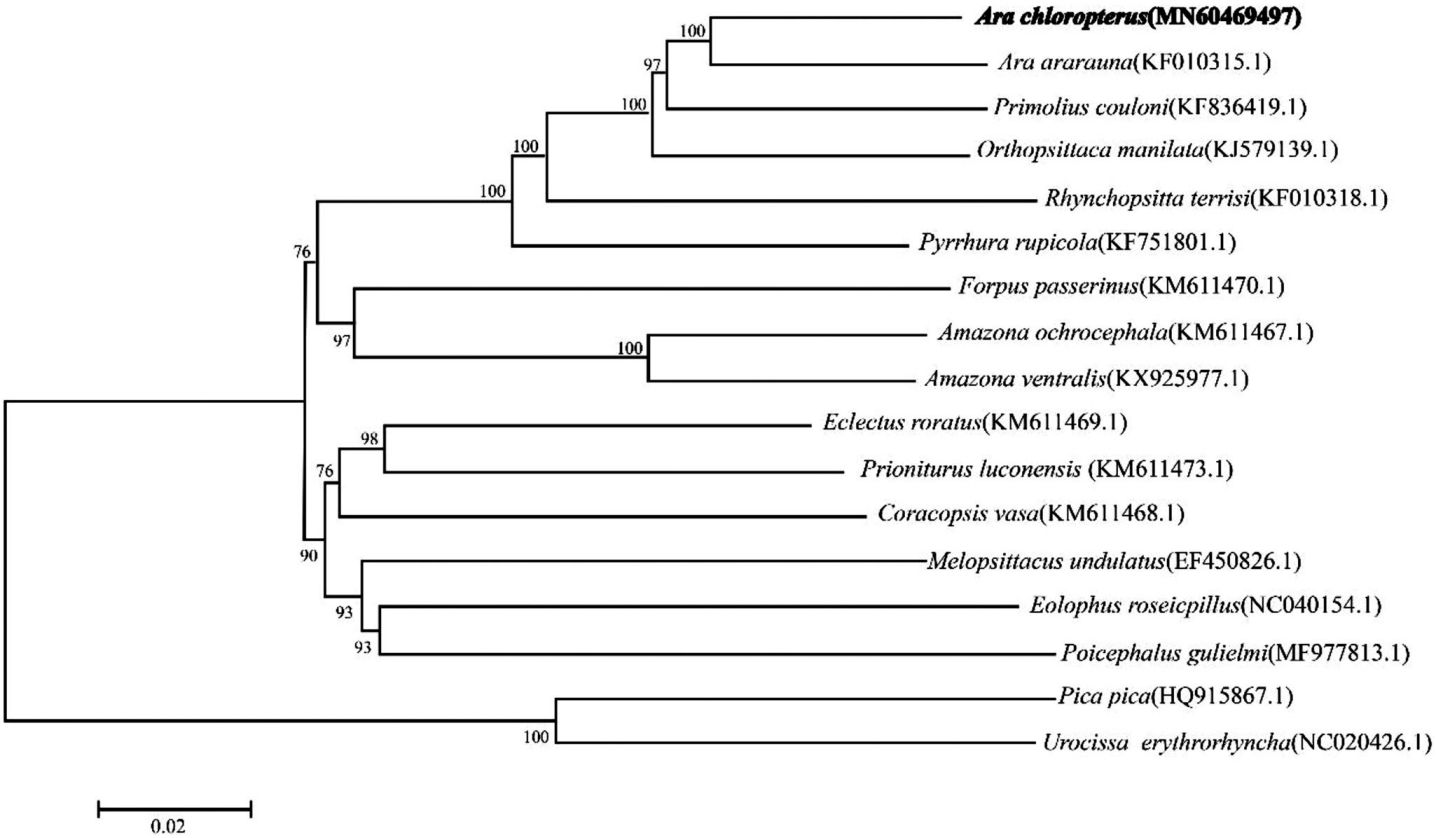
Phylogenetic tree based on the mitogenome of 17 species.

## References

[CIT0001] BirdLife International: Species factsheet: Ara chloropterus. 2019. Cambridge (UK): BirdLife International; [accessed 2019 Oct 30]. http://www.birdlife.org.

[CIT0002] Bridge D, Cunningham CW, Schierwater B, DeSalle R, Buss LW. 1992. Class-level relationships in the phylum Cnidaria: evidence from mitochondrial genome structure. Proc Natl Acad Sci USA. 89(18):8750–8753. 1356268 10.1073/pnas.89.18.8750 PMC49998

[CIT0003] Forshaw JM. 2010. Parrots of the World. London: A and C Black Publishers Ltd.

[CIT0004] Kumar S, Stecher G, Tamura K. 2016. MEGA7: Molecular evolutionary genetics analysis version 7.0 for bigger datasets. Mol Biol Evol. 33(7):1870–1874. 27004904 10.1093/molbev/msw054 PMC8210823

[CIT0005] Lan Y, Liu M, Cao Y. 2019. The complete mitochondrial genome of *Rhinoceros hornbill* (Bucerotiformes: Bucerotidae). Conservation Genet Resour. 11(1):75–78. 12686-017-0972-1.

[CIT0006] Liu DW, Pan Y, Zhou YW, Hou SL. 2019. Complete mitochondrial genome sequence of Nanday conure, *Aratinga nenday* (Psittaciformes: Psittacidae). Mitochondrial DNA Part B. 4(2):3348–3349. 33365987 10.1080/23802359.2019.1673253 PMC7707330

